# Low awareness of lipid disorders amongst individuals despite their high prevalence

**DOI:** 10.1007/s11845-025-03973-w

**Published:** 2025-06-19

**Authors:** Aimee Walsh, Ruth Agar, Greg Offiah, Vincent Maher

**Affiliations:** 1 Advanced Lipid Management and Research (ALMAR) Centre, Tallaght University Hospital, Dublin, Ireland; 2 Department of Cardiology, Tallaght University Hospital, D24 Dublin, Ireland

**Keywords:** Awareness, Lipid disorders, Public health

## Abstract

**Background:**

Atherosclerotic cardiovascular disease (ASCVD) usually goes undetected until complications develop. Knowledge and awareness of cardiovascular risk factors, particularly lipid disorders, lead to earlier disease detection and risk factor intervention.

**Aim:**

We sought to assess the awareness of lipid disorders in a population randomly screened for lipid abnormalities.

**Methods:**

As part of a national awareness campaign, 360 individuals underwent lipid screening and completed a questionnaire regarding their awareness of cardiovascular risk factors.

**Results:**

We identified 240 (66%) individuals with a lipid disorder. Lipid disorders were unrelated to gender but increased sharply from 39% in those under 40 years of age to (71%) in those between 40 and 54 years and (75%) in those older than 55 years. They were more prevalent in those reporting diabetes (78%), hypertension (88%) and hypercholesterolaemia (90%) but not in smokers 62% versus non-smokers 68%. Awareness of abnormal lipids was present in only 34% of individuals overall but was higher in those with diabetes (70%) hypertension (64%) or in those with two or more risk factors (50%). Awareness of lipid disorders increased with age, being extremely low (8%) in those under 40 years of age and increasing to (28%), (45%) and (54%) in those aged 40 to 54 years, 55 to 70 years and older than 70 years, respectively. The biggest gap between the presence and awareness of lipid disorders occurred in the 40 to 54 year age group. There were significant correlations between the awareness of lipid disorders and the awareness of hypertension *r* = 0.27, *p* < 0.01 or diabetes *r* = 0.14, *p* < 0.05 but not of smoking *r* =  − 0.04 or family history of ASCVD *r* = 0.11.

**Conclusions:**

Given the importance of early detection and intervention to reduce cardiovascular disease risk factors, public education and increased screening are advisable, especially for lipid disorders in the youngerage groups.

## Introduction

Atherosclerotic cardiovascular disease (ASCVD) develops from infancy, and individuals are usually unaware of its progression until complications such as angina, myocardial infarction or stroke ensue [1]. Epidemiological studies have confirmed strong associations between ASCVD and risk factors such as diabetes [2], hypertension [3], smoking [4], obesity [5] and particularly lipid disorders [6] [7] [8]. As these risk factors usually have no associated symptoms, screening is unlikely unless awareness is present [9, 10]. Awareness would enhance the likelihood of disease detection and earlier risk factor intervention to prevent ASCVD complications [10, 11].

Awareness requires education, and it is therefore important to ascertain how much awareness currently exists to see what gaps in knowledge are present to help target future public education programmes. Given that lipid disorders are particularly important in ASCVD development [7, 12], we sought to determine the awareness of lipid disorders in a randomly screened population.

Amongst lipid disorders, raised low-density lipoprotein (LDL) cholesterol levels are causally linked with cardiovascular disease [7]. Raised triglyceride levels have a variable relationship with atherosclerosis, with intermediate levels being associated with cardiovascular disease [12] [8]. Low levels of HDL cholesterol are associated with increased ASCVD risk ([13]).

Based on accepted abnormal lipid levels [14], we sought to identify those with a lipid abnormality and from an associated questionnaire, we endeavoured to determine their awareness of same.

### Methodology

The aim of this study was to assess awareness of lipid abnormalities in subjects who were screened randomly in the community and in a hospital setting. Subjects who underwent non-fasting cholesterol screening [15] in five different rural locations around Ireland as part of a national cholesterol awareness campaign and others at a city hospital screening day had cholesterol testing and completed a detailed questionnaire regarding cardiovascular risk factors.

The questionnaire included age, gender, family history of cholesterol disorders or heart disease, use of lipid-lowering medications, presence of other cardiac risk factors, smoking status and alcohol intake per week.

The lipid testing was done using a Cholestech LDX machine by obtaining finger-tip blood samples. The samples were analysed in 5 min to show the results of total cholesterol, triglyceride, HDL cholesterol, LDL cholesterol (Calculated by the Freidewald equation) and non-HDL cholesterol. The Cholestech LDX machine had been previously validated by us to correspond accurately to laboratory values [16].

Reference values for non-fasting lipid samples were derived from ESC guidelines for cardiovascular disease prevention [15][14] with the following levels applied as being abnormal: total cholesterol > 5.0 mmol/l, triglyceride > 2.0 mmol/l, HDL cholesterol < 1.0 for men and 1.2 mmol/l for women, LDL cholesterol > 3.0 mmol/l, non-HDL cholesterol > 3.9 mmol/l. The limitation of the Cholestech machine is that HDLc values > 2.59 or triglyceride values < 0.51 were not measurable [16].

Each subject discussed their results with a consultant or specialist nurse, and actions were advised, including review by their general practitioners if necessary. All subjects agreed to their anonymised data being included in a publication.

#### Participant consent and ethics

Ethical approval for this study was achieved from Trinity College Dublin ethics committee. Following informed consent, subjects who volunteered at the various testing sites filled in the questionnaire and underwent near point lipid testing.

#### Statistical analysis

All data were analysed using SPSS version 29 software. Descriptive statistics were used to define the total population and the various subgroups. ANOVA was used to compare awareness levels in the various subgroups with lipid abnormalities.

## Results

The demographics of the 360 subjects who underwent the screening in the community and in the hospital are outlined in Table 1. There was a high prevalence of lipid abnormalities (66%) which was not gender-specific but did increase significantly with age, particularly after the age of 40 years, when it was present in over 70% of the participants. Those reported personally having cardiovascular disease had a higher prevalence (76%) of lipid abnormalities. Those who reported personally having any of the traditional cardiovascular risk factors, e.g. family history of CVD (70%), diabetes (78%), hypertension (88%) and hypercholesterolaemia (90%), had a higher prevalence of lipid abnormalities. In addition, those who reported a family history of hypercholesterolaemia had also a significantly higher prevalence of lipid abnormalities (66%). Of note, those who reported not having any of the traditional risk factors still had a very high prevalence (61%) of lipid abnormalities. The prevalence of lipid abnormalities did increase in proportion to the number of traditional cardiovascular risk factors, with 74% of those with two or more risk factors having a lipid disorder. It was also noted that there was a similar high prevalence of lipid abnormalities in those screened in either the community (65%) or the hospital (70%).

**Table 1:** Demographics of patient population

		Total	Normal lipids	Abnormal lipids
*n*		360	120 (33%)	240 (66%)
Male		127	37 (29%)	90 (71%)
Female		233	83 (36%)	150 (64%)
Age, years *n* = 356	< 40	82	50 (61%)	32 (39%)
	40–54	116	32 (28%)	84 (72%)
	55–70	124	28 (23%)	96 (77%)
	> 70	34	8 (21%)	26 (79%)
Known personal CVD	Yes	17 (5%)	4 (24%)	13 (76%)
	No	317 (88%)	104 (33%)	214 (67%)
	?	26 (7%)	12 (48%)	13 (52%)
Fam Hx CVD	Yes	159 (44%)	47 (30%)	112 (70%)
	No	158 (44%)	58 (37%)	100 (63%)
	?	43 (12%)	15 (35%)	28 (65%)
Diabetes	Yes	9 (3%)	2 (22%)	7 (78%)
	No	124 (34%)	44 (35%)	80 (65%)
	?	227 (63%)	74 (33%)	153 (67%)
Hypertension	Yes	41 (11%)	5 (12%)	36 (88%)
	No	110 (31%)	42 (38%)	68 62%)
	?	209 (58%)	72 (35%)	135 (65%)
Hypercholesterolaemia	Yes	80 (22%)	8 (10%)	72 (90%)
	No	98 (27%)	37 (38%)	61 (62%)
	?	182 (51%)	75 (41%)	107 (59%)
Smoking	Yes	39 (11%)	15 (38%)	24 (62%)
	No	289 (80%)	93 (32%)	196 (68%)
	Ex	32 (9%)	12 (38%)	20 (62%)
Fam Hx high chol	Yes	124 (34%)	42 (34%)	82 (66%)
	No	112 (32%)	41 (37%)	71 (63%)
	?	124 (34%)	37 (30%)	87 (70%)
Risk factors	0	141	55 (39%)	86 (61%)
	1	163	50 (31%)	113 (69%)
	> 2	54	14 (26%)	40(74%)
Hospital screening	*n*	101 (28%)	30 (30%)	71 (70%)
Community screening	*n*	259 (72%)	90 (35%)	169 (65%)

**Fig. 1:**
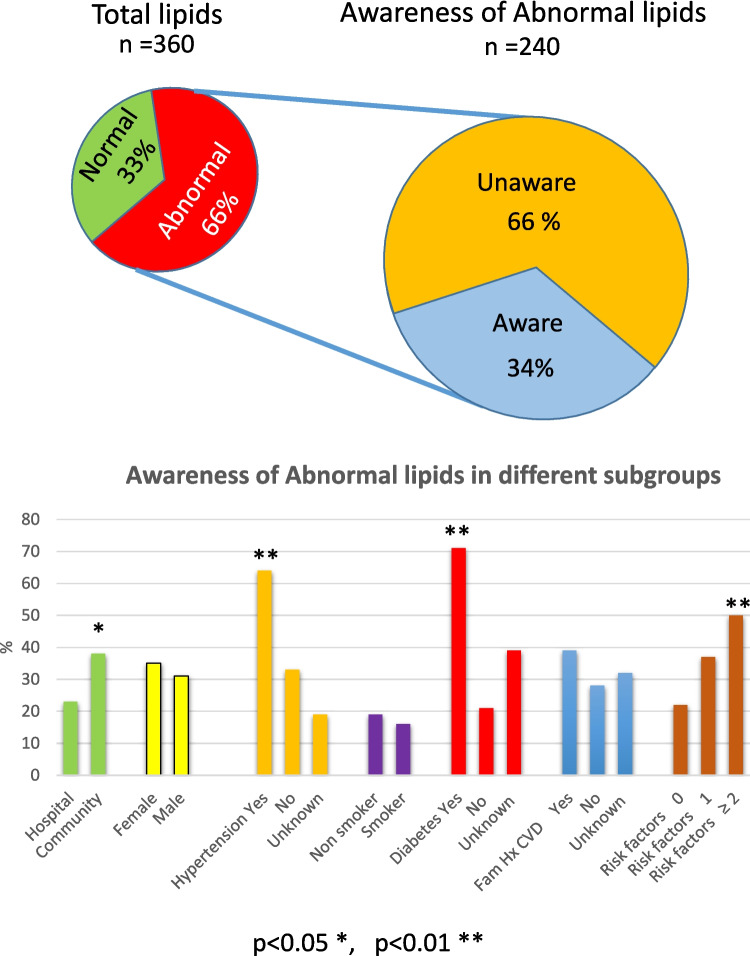
Prevalence and awareness of lipid disorders

Figure 1 outlines the prevalence and awareness of lipid abnormalities. It is evident that two thirds of those screened had a lipid abnormality. Of those with a proven lipid abnormality, just over one third (34%) were aware of their lipid abnormality. Awareness was not different between men and women but was higher in those who were screened in the community (37%) compared to those screened in the hospital setting (24%) *p* < 0.05. The awareness level was higher in those with hypertension (64%) and diabetes (71%) but not in those with a reported family history of cardiovascular disease or in those who smoked. The awareness did increase in proportion to the number of cardiovascular risk factors reported, reaching 50% in those reporting two or more cardiovascular risk factors.

**Fig. 2:**
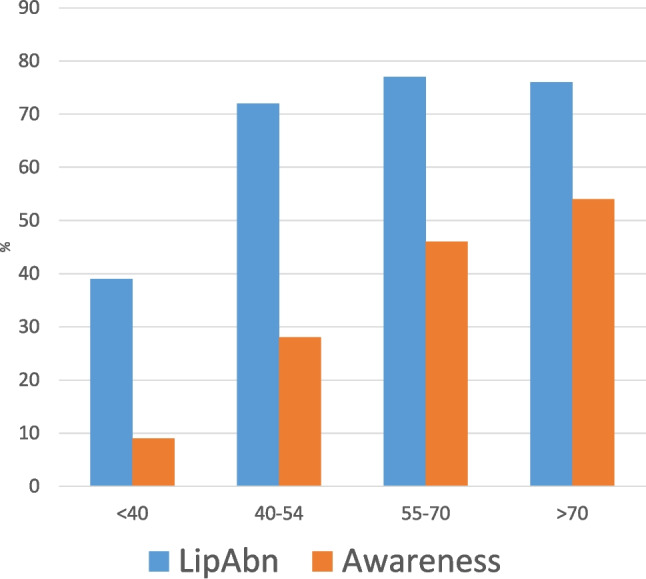
Lipid abnormality prevalence versus awareness according to age range

Figure 2 outlines the prevalence and awareness of lipid abnormalities according to the subjects’ age. There was a marked increase in the prevalence of lipid abnormalities after the age of 40 years, i.e. 38% in those under 40 years and exceeding 70% in all those older than 40 years. Although the level of awareness did increase with age, there was an obvious lag in the awareness in the 40 to 54-year age group. The average age of those who were unaware of their lipid abnormality was 51 ∓ 13 years. The average LDL cholesterol in this group was 3.2 ∓ 0.9 mmol/l.

## Discussion

This study highlights that there is a low level of awareness of lipid disorders (only 34%) despite their high prevalence (66%) in this moderate cardiovascular disease risk population [14]. This is not too dissimilar to the result in a much larger population from Eastern Asia where awareness was 40% [17, 18] However, in this study, the criteria for the presence of abnormal lipids was much higher with LDLc levels > 4.1 mmol/l and triglycerides > 2.3 mmol/l, and the prevalence was thus only 28.4%. The lipids were measured fasting in subjects selected by a stratified random cluster sampling. Nonetheless, like in our study, abnormal lipid prevalence was higher in those with diabetes and hypertension. The level of awareness was also related to increasing age and the presence of diabetes, hypertension and level of education, although the latter was not assessed in our study. This is not surprising given the cumulative effect of additional risk factors on CV outcomes from population studies such as Framingham. Once one risk factor is identified, the search for others may become more automatic or, as a consequence of having one risk factor, individuals spend more time in a medical environment where testing is undertaken.

Compared to a longitudinal study of Swiss subjects screened annually, awareness levels in our study were much lower at 33% vs 77.3% [18]. However, the prevalence of hyperlipidaemia was much higher in our population compared to the Swiss at 66% vs 35%. This might be explained by the assignment of dyslipidaemia to just raised LDLc and lipid lowering treatment in the Swiss. When we analysed our patients with just these criteria, the awareness rose to 50%.

It is interesting that there was also a low level of awareness of hypertension and diabetes in our population. There was a strong correlation between the level of awareness of these three risk factors, which argues that it is not just the type of risk factor but the lack of education or concern regarding cardiovascular risk factors in general.

Awareness did increase with age, which is similar to the findings in the large Chinese population [17] but there was a lag in the prevalence of awareness level in the 40 to 54-year age group in our population despite the markedly increased prevalence of lipid disorders. In addition, we did not note a gender difference in awareness in our population unlike the higher awareness in Chinese females.

The level of awareness was greater in those screened in the community than in the urban hospital which is slightly surprising given the greater number of medical professionals in the hospital setting. This contrasts with the findings in the Chinese study, where awareness in the urban setting was significantly greater than in the community. This may be just a chance finding in our study or coincide with the younger age range of those screened in the urban hospital setting. Different access to better education and healthcare in Chinese urban vs community areas may have a bearing on these observations, which may not be the case in Ireland.

The impact of lack of awareness for individuals with persistently raised cholesterol levels will lead inevitably to an increased cardiovascular risk in proportion to the high untreated LDLc levels [7]. This cumulative risk will also relate to the number of years abnormal lipids remain undiagnosed. In this regard, the average age of those unaware of their risk factors was 51 ∓ 13 years and the average LDL cholesterol was 3.2 ∓ 0.9 mmol/l. Their average age and level of LDL cholesterol do not inform us about the cumulative LDL cholesterol exposure but do highlight unawareness of the potential high exposure to abnormal lipids during the greater part of an individual’s lifespan.

There are a number of limitations in this study. It has small numbers and people were invited to participate. There was therefore a potential bias towards those more concerned about their ASCVD risk than in the large randomly selected Chinese population [17]. Consequently, it is not representative of a national Irish population. Completion of questionnaires was only over a short period of time when cholesterol screening was undertaken in a variety of locations such as a community hall, hotel, shopping centre, hospital, construction company workplace and a public house. More time and assistance with completion of the questionnaires may have yielded different results. However, allowing for these potential limitations impacting the results to an extent, it is unlikely that these results do not signify a particularly high level of unawareness concerning the presence of a lipid disorder.

These findings have an important public health consequence given the accepted causal relationship between lipid disorders and ASCVD. In this regard, action needs to be undertaken to screen and treat individuals much earlier in their lifespan. It is now advised that all children should be screened for lipid disorders notable FH at a young age [19]. This would also allow earlier detection of other significant forms of lipid disorder. It is therefore important that education of health care workers is increased and that screening programmes become more widespread. Ultimately, a policy needs to be implemented to ensure every individual is at least aware and educated so that long term they can make informed decisions about early intervention of potential cardiovascular risk factors, particularly lipid disorders.
